# Supplementary material for: Hematophagy and tick-borne Rickettsial pathogen shape the microbial community structure and predicted functions within the tick vector, Amblyomma maculatum
Source: Front Cell Infect Microbiol. 2022 Nov 21;12:1037387. doi: 10.3389/fcimb.2022.1037387 (PMC9719966; doi:10.3389/fcimb.2022.1037387)
Supplement: Supplementary Figure 3 — Stability of bacterial abundances across developmental stages. The relative abundances of the predominant members of microbial communities in (A) all developmental stages, (B) unfed and fully fed developmental stages and (C) R. parkeri-infected and uninfected developmental stages. Each bar on the abundance plot represents average data from 3–5 individual replicates. Unfed (UF), fed (F), larvae (LV), nymph (N), male (M), female (F), clean (CL), infected (IN). [file DataSheet_1.docx]

**ADDITIONAL FILES**


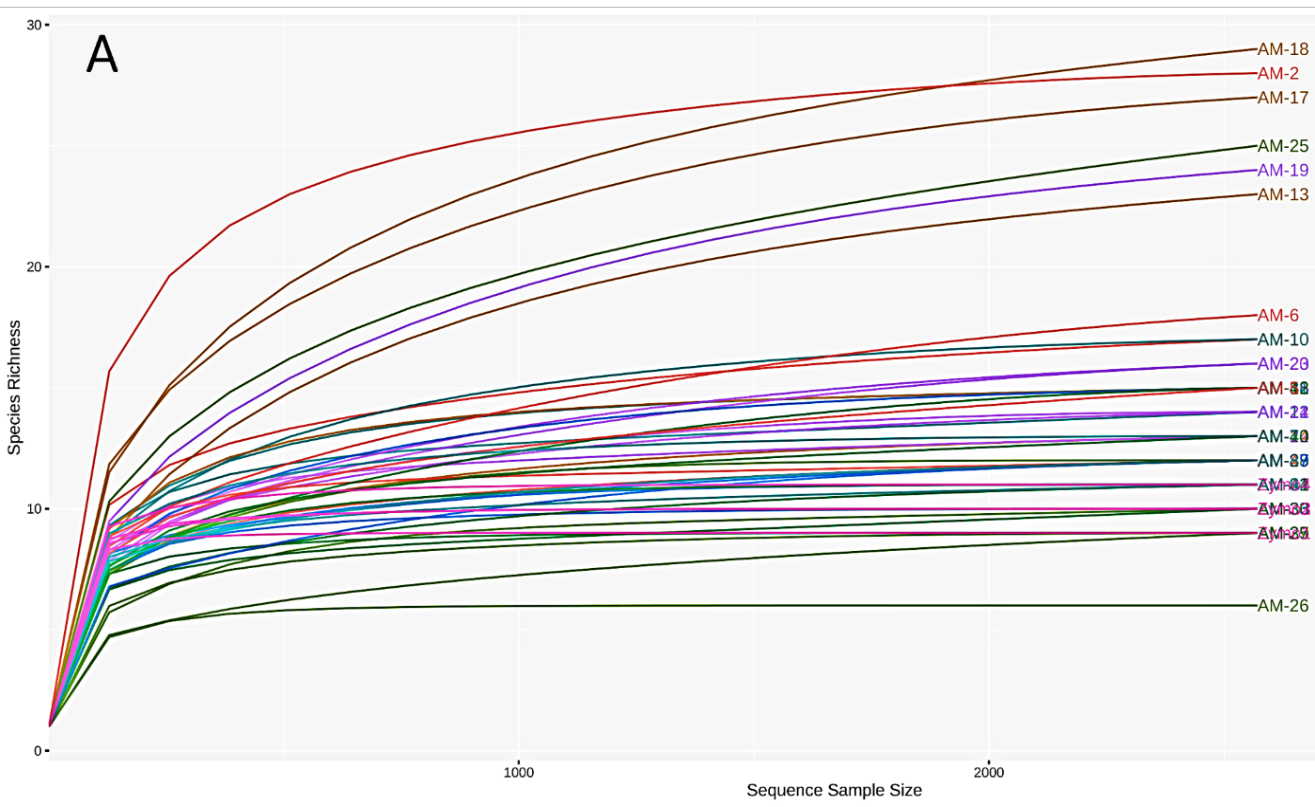

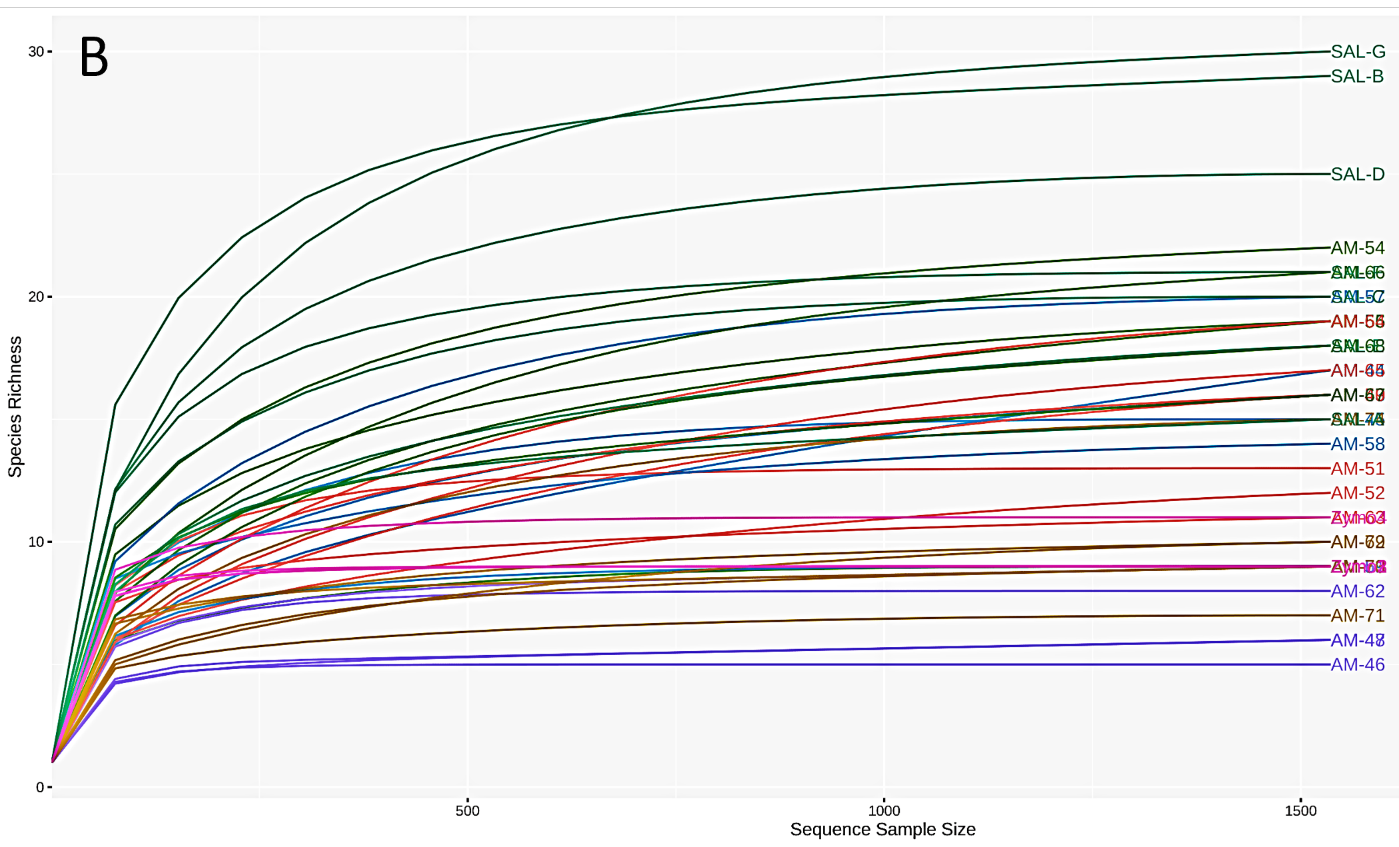


FIGURE S1: **Rarefaction analysis of raw reads** from A) developmental stages and B) isolated tissues of *Am. maculatum* ticks. Each curve representing individual replicates was rarified to a sequence depth of approximately 3000 sequences for all developmental stages (unfed and partially fed) and 1000 sequences for isolated tissues.


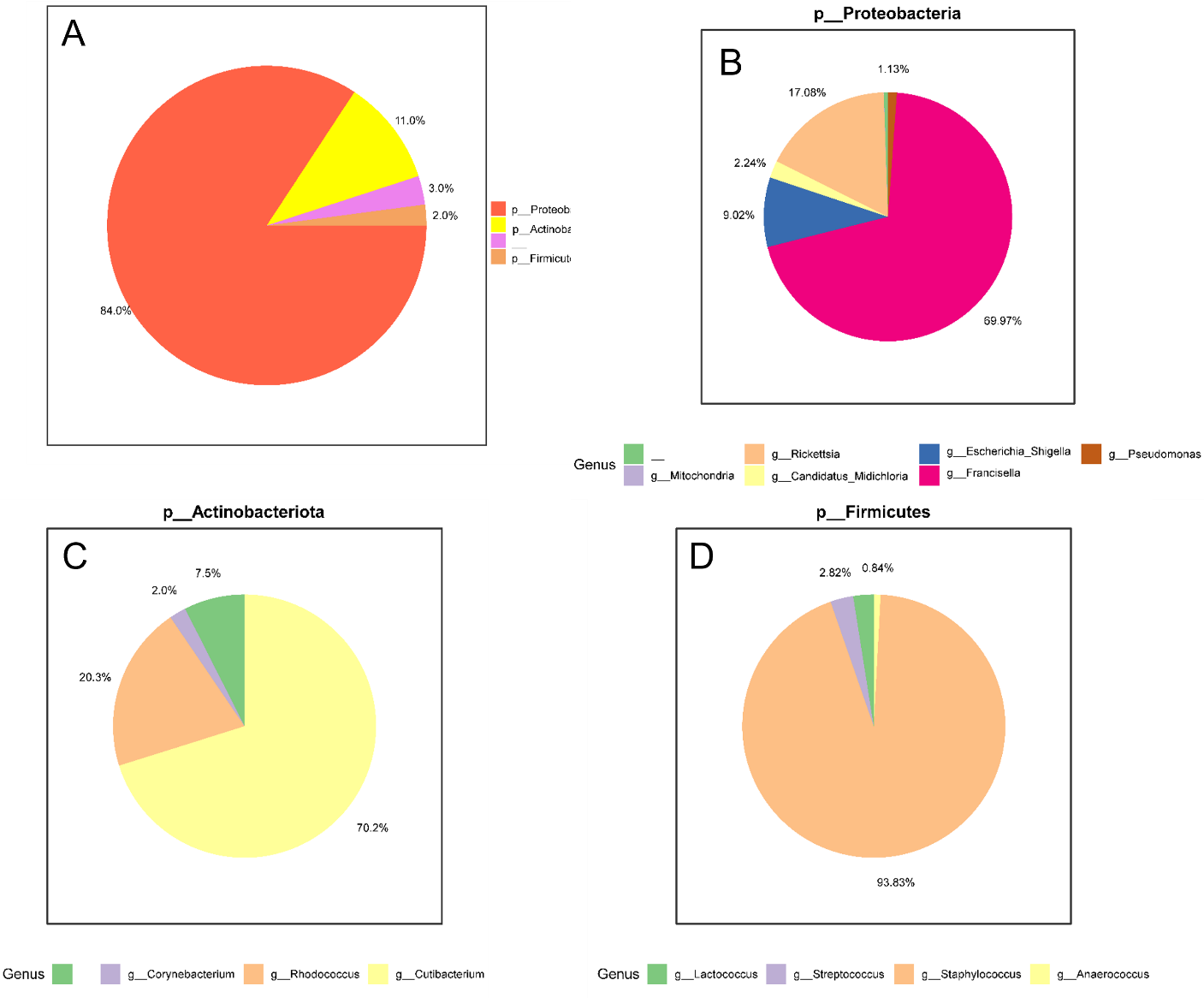
FIGURE S2**: Pie chart summary of microbial abundances in the developmental stages of Am. maculatum.** A) Overall abundance summary at the phylum taxonomic level and breakdown of abundant phyla showing B) Proteobacteria, C) Actinobacteriota, and D) Firmicutes. A representative genus from each phylum is indicated in the legends.

FIGURE S3: **Stability of bacterial abundances across developmental stages.** The relative abundances of the predominant members of microbial communities in A) all developmental stages, B) unfed and fully fed developmental stages and C) *R. parkeri*-infected and uninfected developmental stages. Each bar on the abundance plot represents average data from 3–5 individual replicates. Unfed (UF), fed (F), larvae (LV), nymph (N), male (M), female (F), clean (CL), infected (IN).


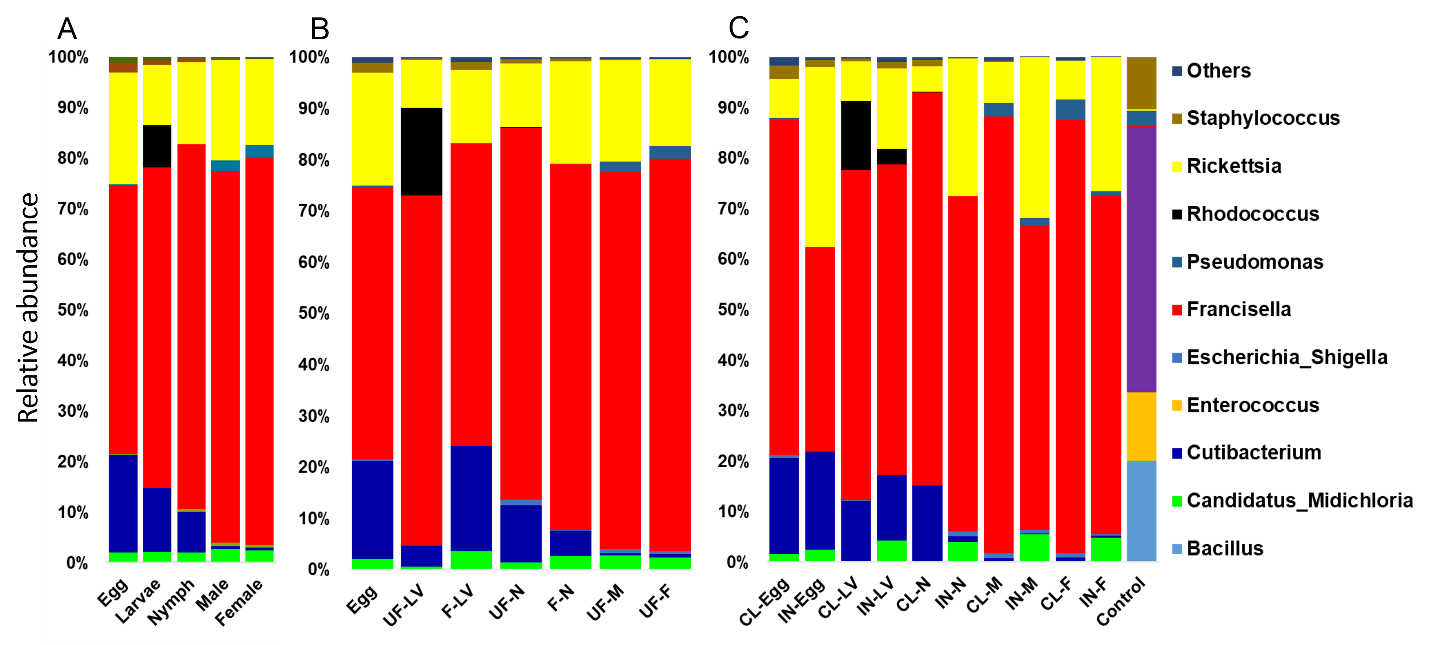


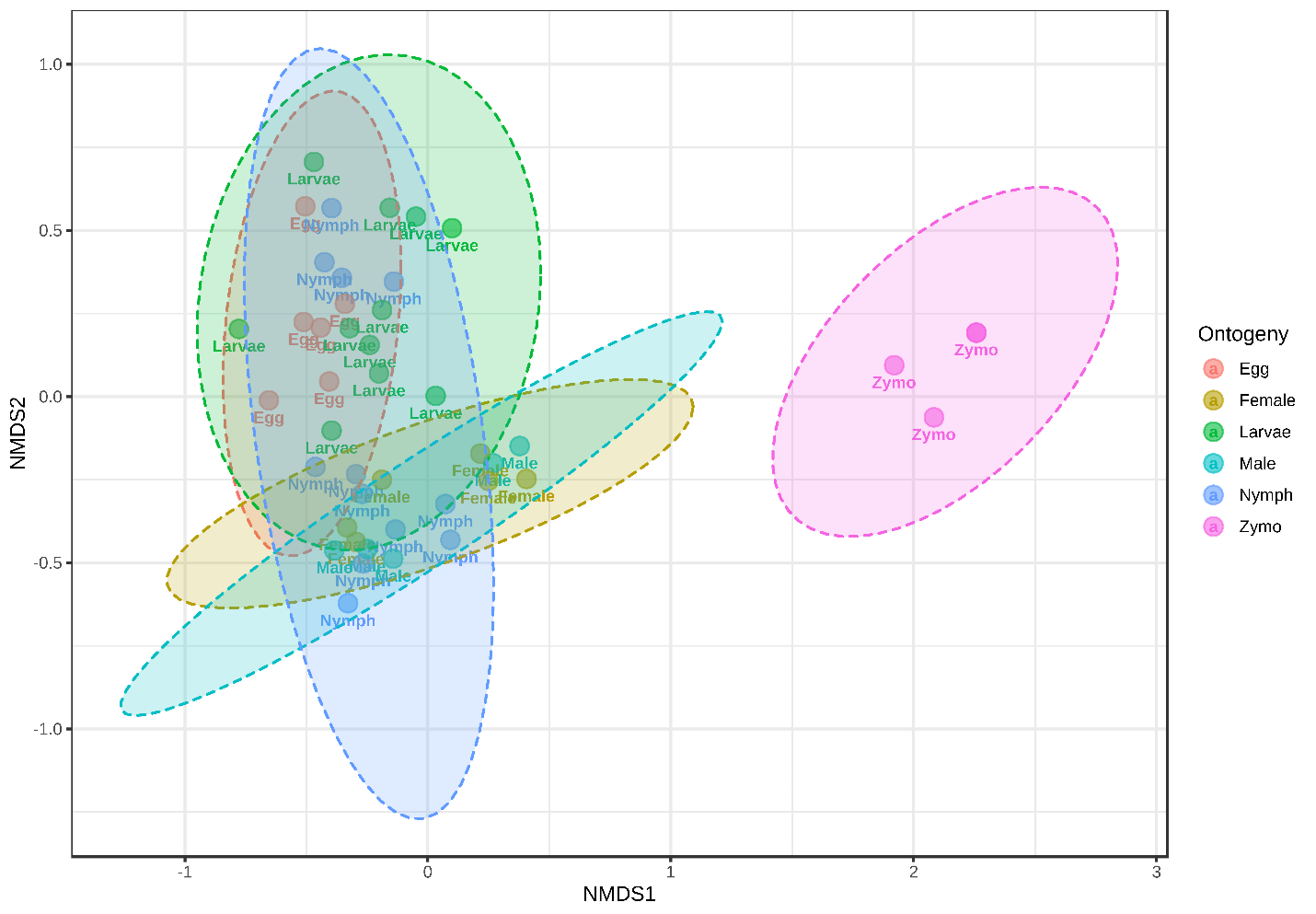


FIGURE S4**: Non-metric multidimensional scaling (NMDA) analysis of** β-diversity measures across ***Am. maculatum* developmental stages.** Ellipses around unique clusters indicate the degree of distance based on the Bray–Curtis distance matrix.


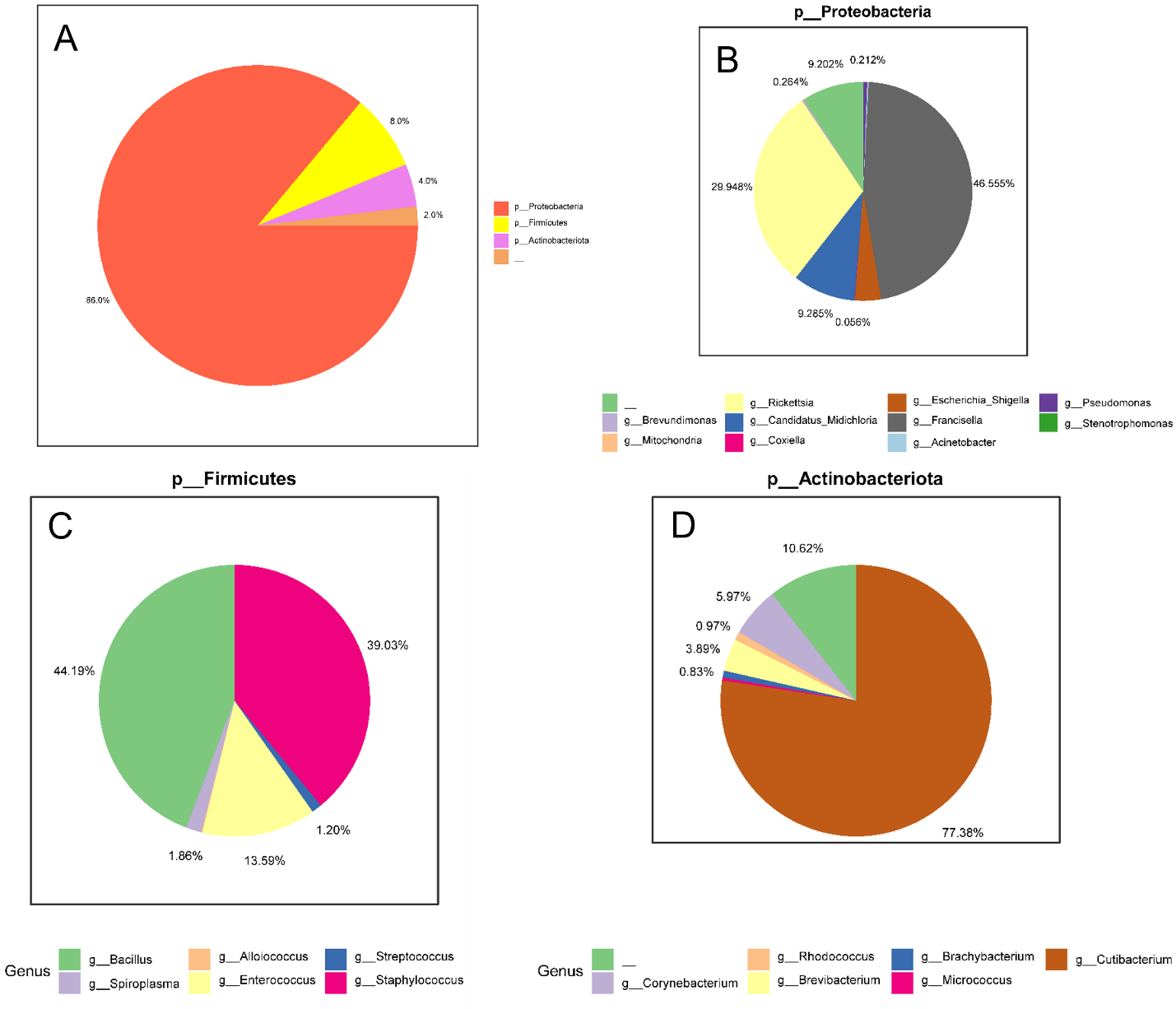


FIGURE S5: **Pie chart summary of microbial abundances in the dissected tissues of *Am. maculatum*.** A) Overall abundance summary at the phylum taxonomic level and breakdown of abundant phyla showing B) Proteobacteria, C) Firmicutes, and D) Actinobacteriota. A representative genus from each phylum is indicated in the legends.

FIGURE S6: **Changes in microbial assemblages and diversity across different tissues.** The relative abundances of the predominant members of microbial communities in A) all isolated tissues, B) unfed and fully fed tissues, and C) *R. parkeri*-infected and uninfected tissues. Each bar on the abundance plot represents average data from 3–5 individual replicates. Unfed (UF), fed (F), larvae (LV), nymph (N), male (M), female (F), clean (CL), infected (IN), salivary gland (SG), midgut (MG), ovary (OV).


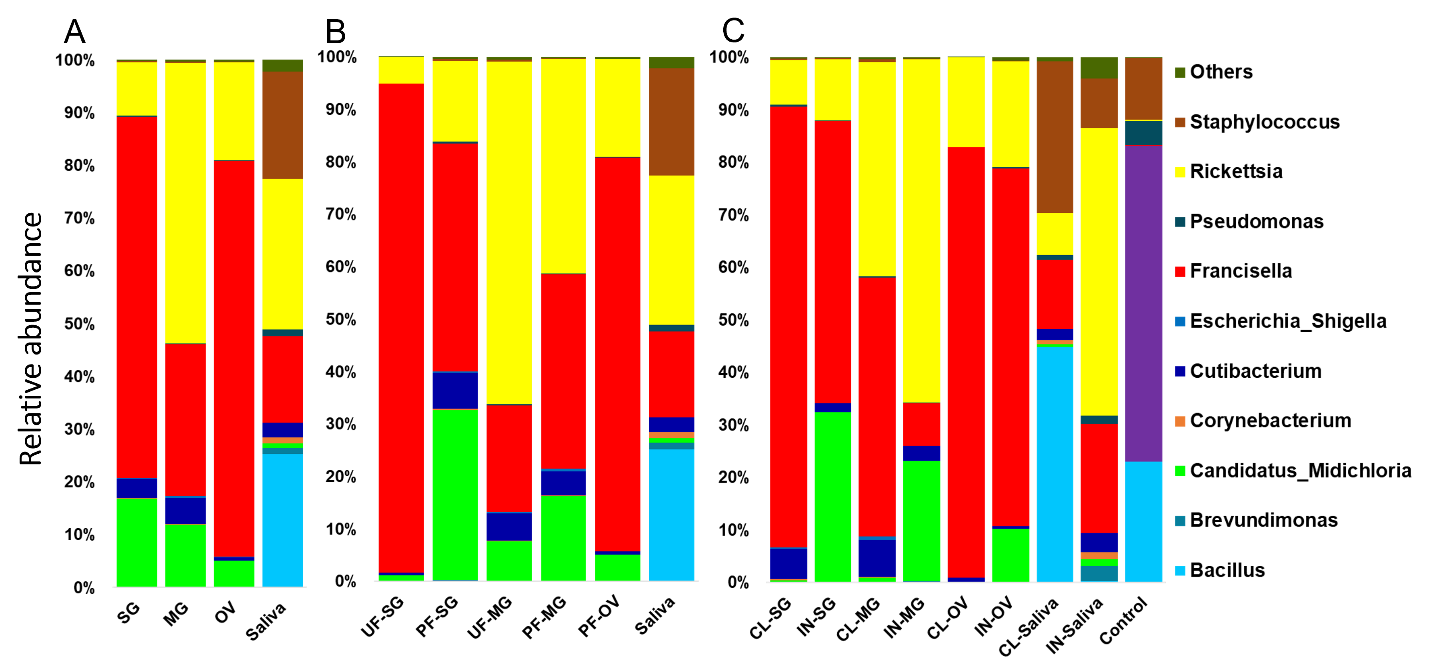


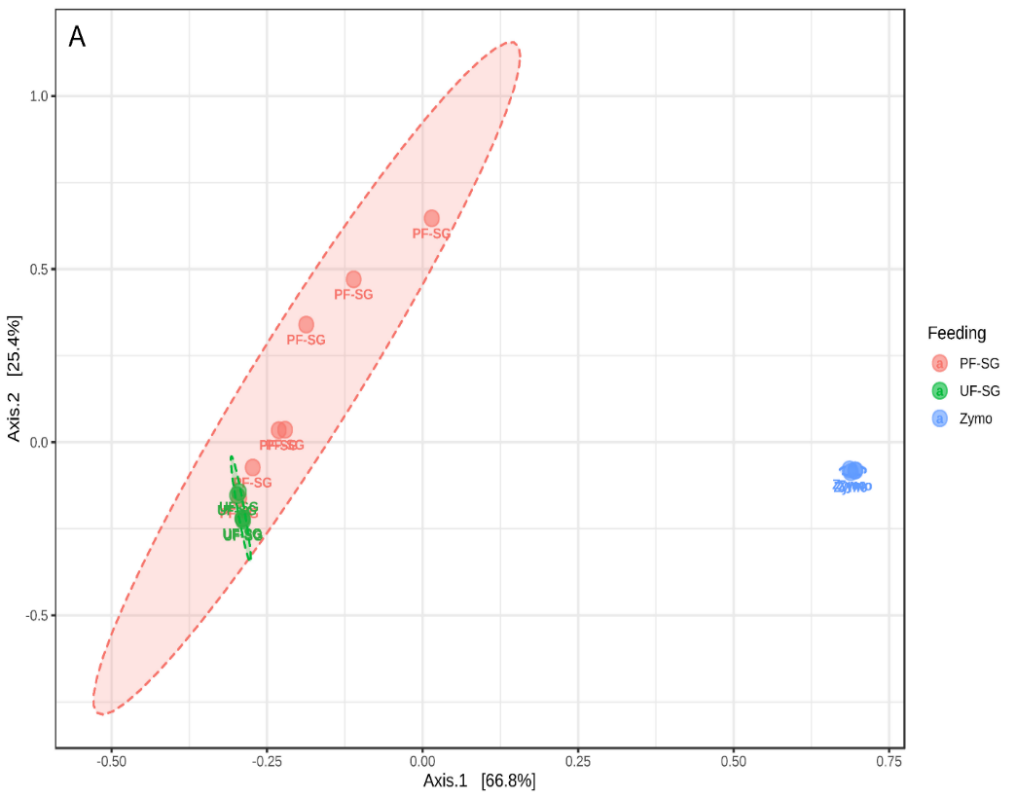

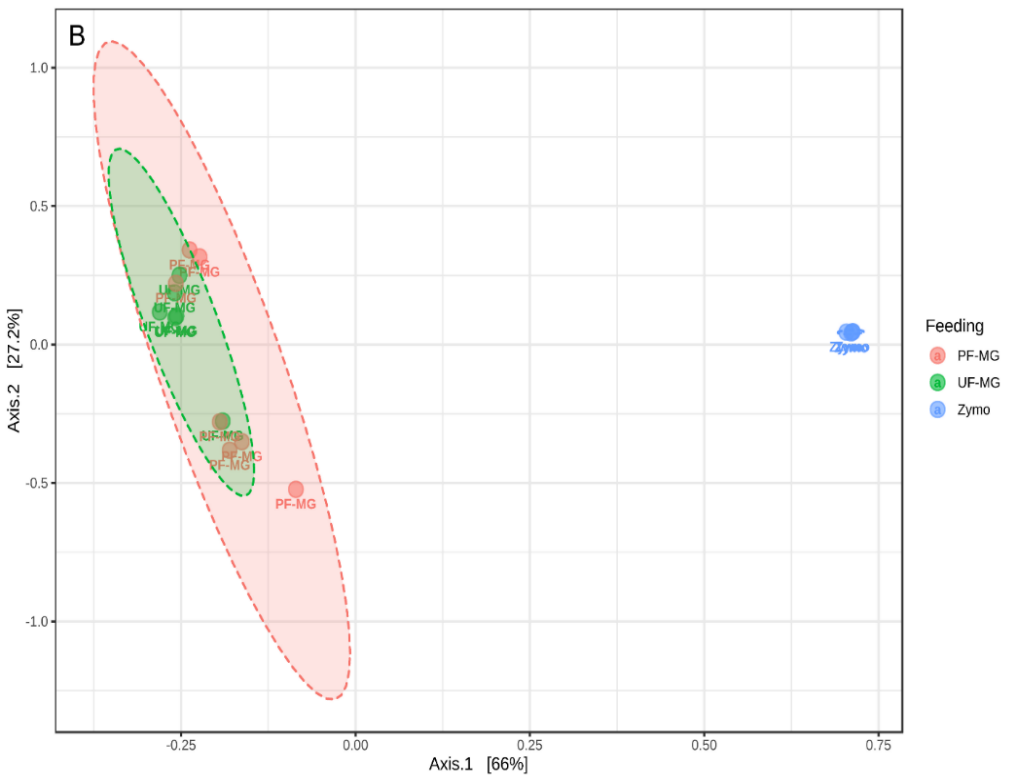


FIGURE S7**: Principal coordinate analysis of β-diversity measures of tissues dissected from unfed and partially fed *Am. maculatum* ticks.** Bray–Curtis distance matrix of A) salivary gland and B) midgut tissues. Ellipses around unique clusters indicate the degree of distance.


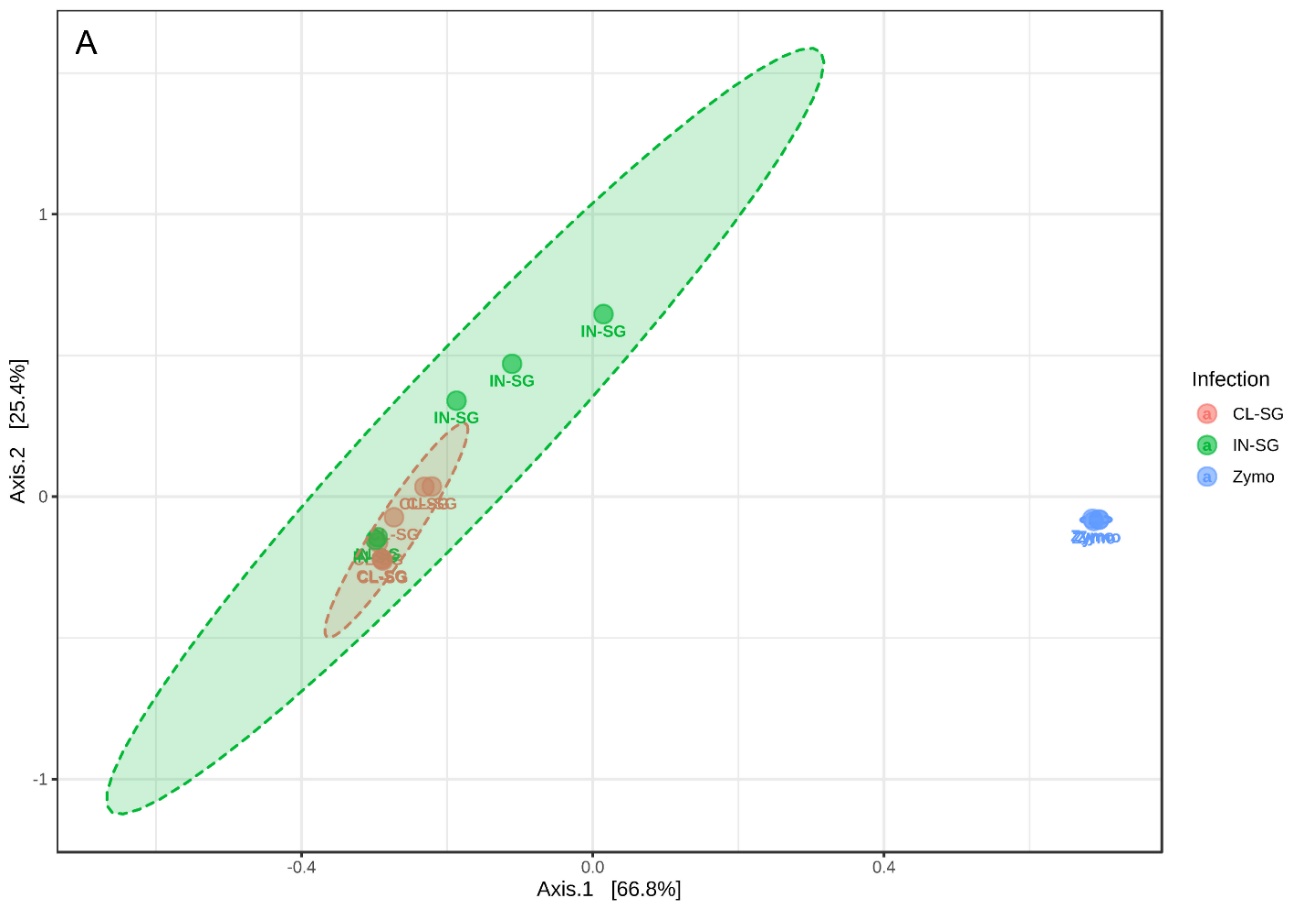

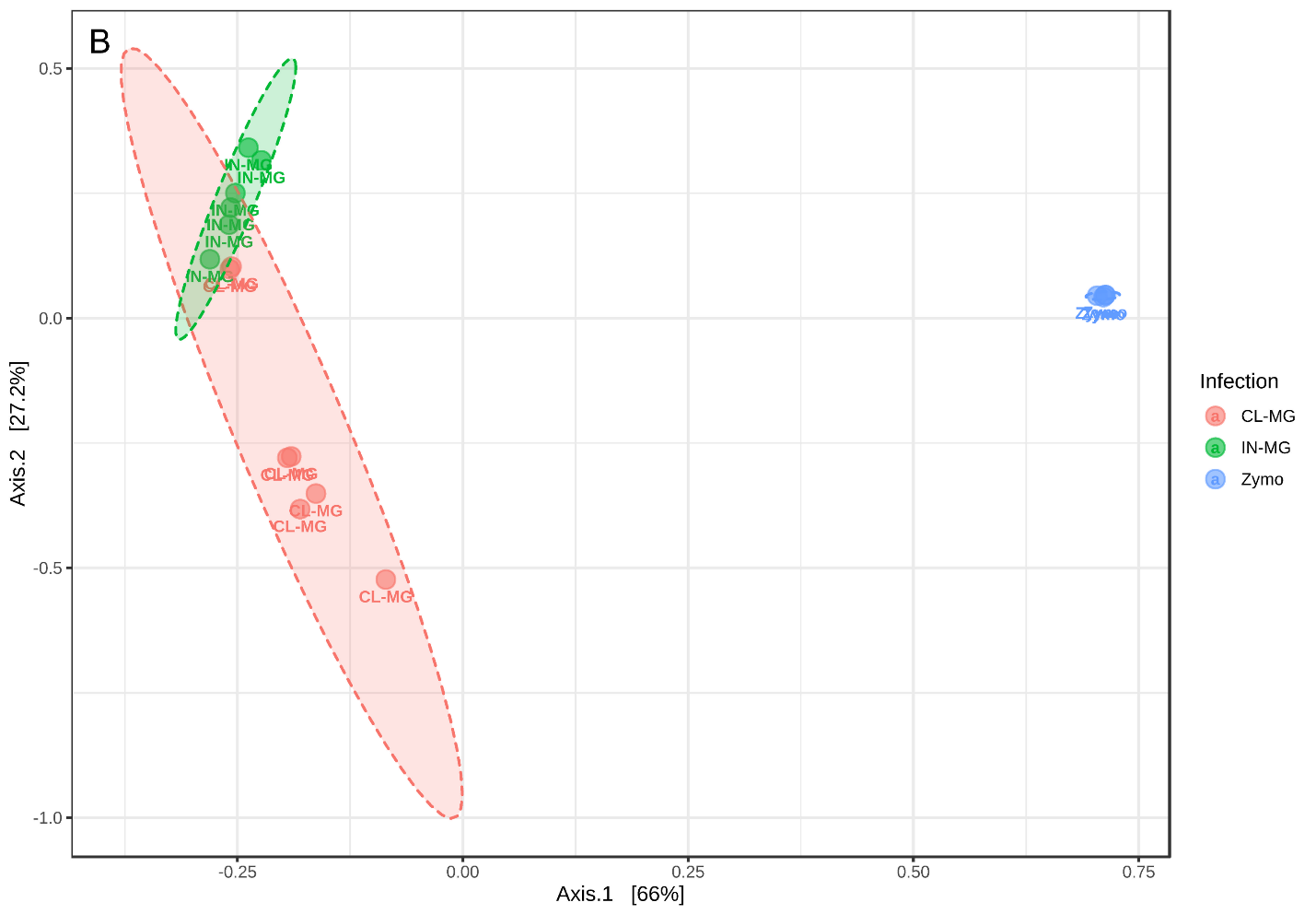


FIGURE S8**: Principal coordinate analysis of β-diversity measures of tissues dissected from unfed and partially fed *Am. maculatum* ticks.** Bray–Curtis distance matrix of A) salivary gland, B) midgut tissues, and C) ovarian tissues. Ellipses around unique clusters indicate the degree of distance.


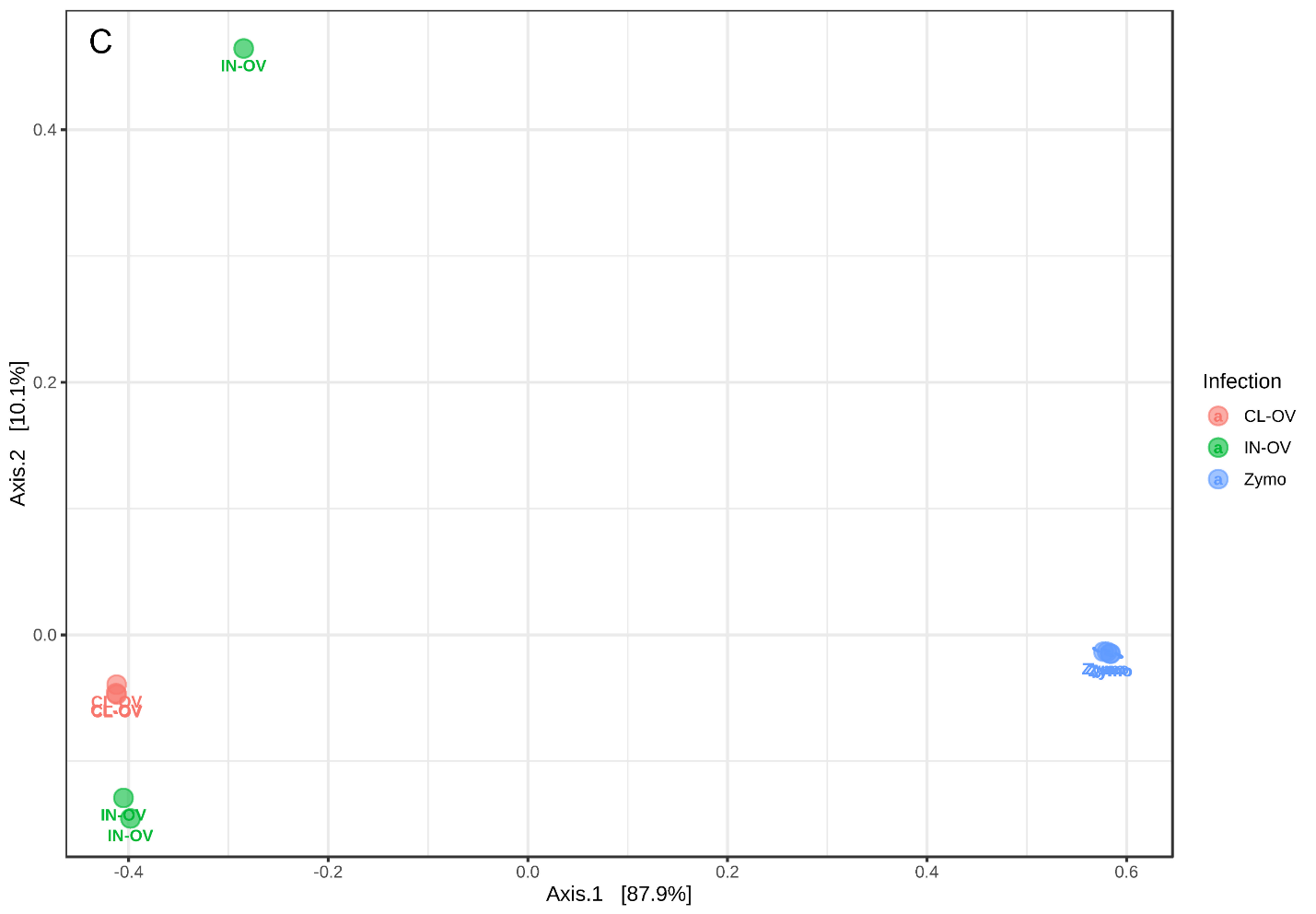


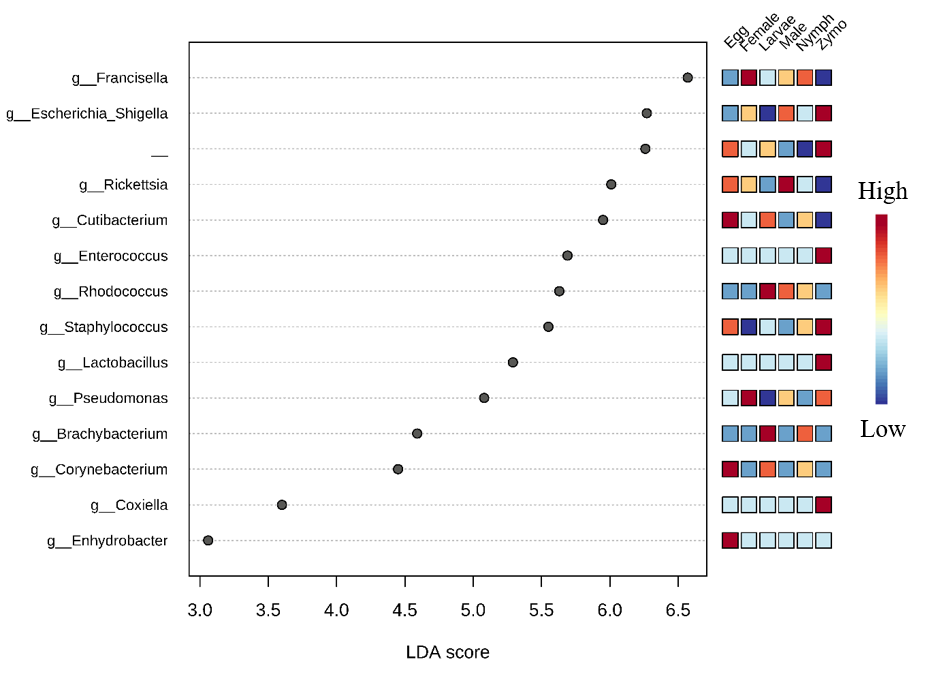


FIGURE S9**: Graphical summary of LEfSe analysis across the developmental stages of *Am. maculatum*.** Significant taxa are ranked in decreasing order by their LDA scores (x axis). The mini heat map to the right of the plot indicates whether the number of taxa is higher (red) or lower (blue) in each group.


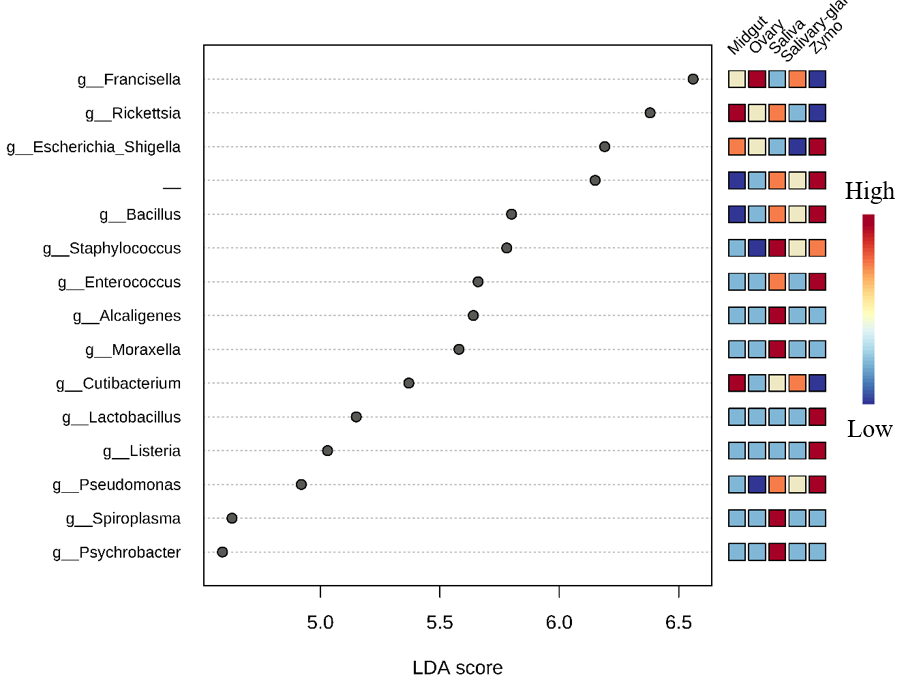


FIGURE S10**: Graphical summary of LEfSe analysis from the dissected tissues of *Am. maculatum*.** Significant taxa are ranked in decreasing order by their LDA scores (x axis). The mini heat map to the right of the plot indicates whether the number of taxa is higher (red) or lower (blue) in each group.

FIGURE S11**: Graphical summary of LEfSe analysis from the dissected tissues of *Am. maculatum*.** A) *R. parkeri*-infected and uninfected and B) unfed and partially fed *Am. maculatum*. Significant taxa are ranked in decreasing order by their LDA scores (x axis). The mini heat map to the right of the plot indicates whether the number of taxa is higher (red) or lower (blue) in each group.


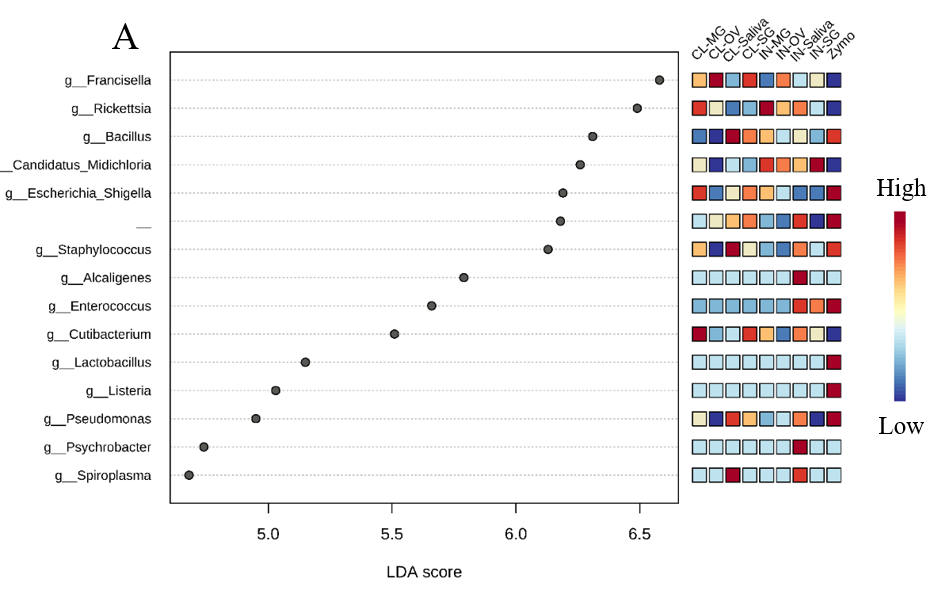

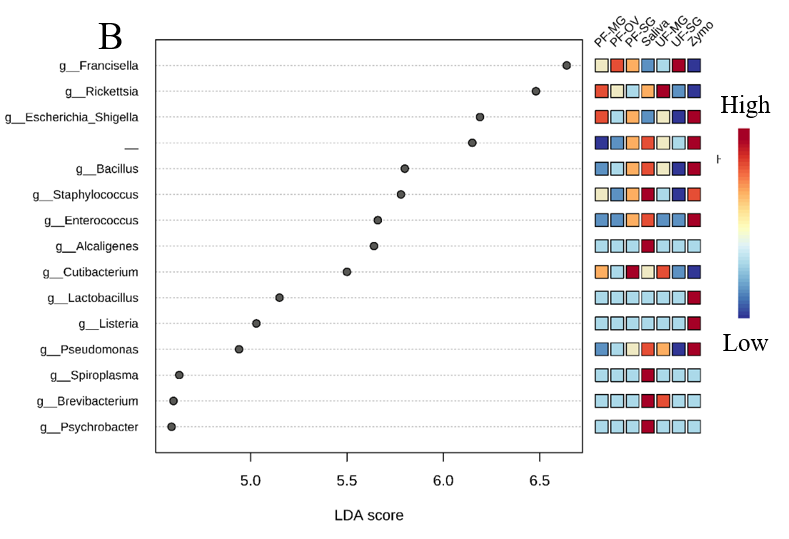


**
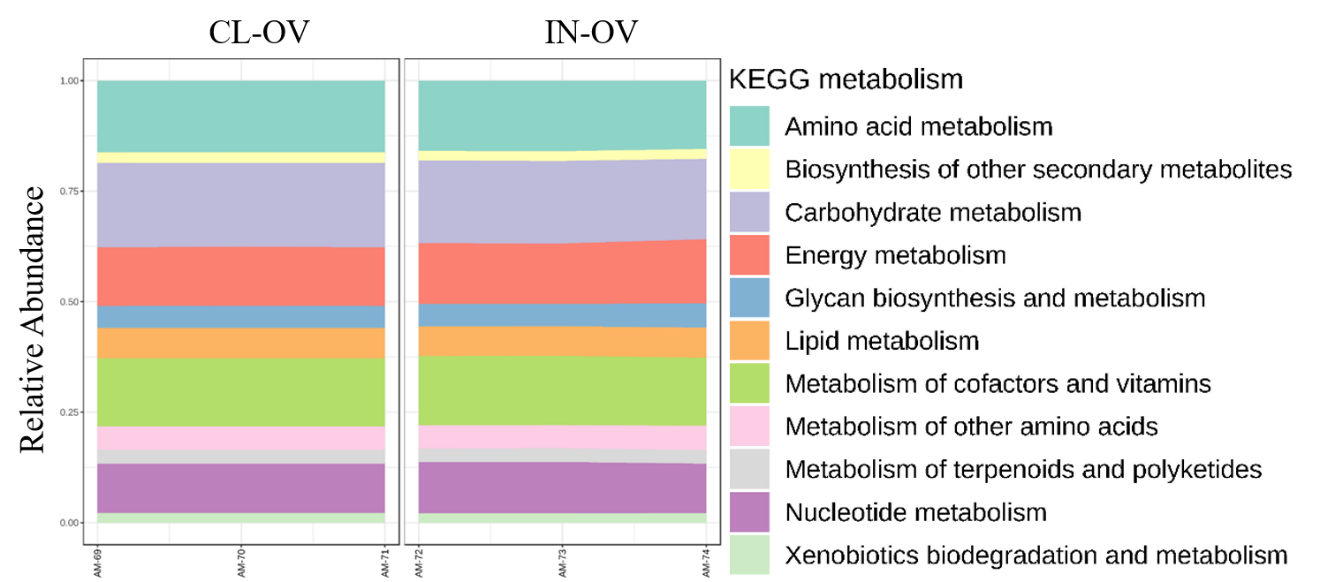
**FIGURE S12: **Histogram indicating functional differences of the Am. maculatum microbiota** across developmental stages. KEGG metabolic categories were obtained from 16S rRNA gene sequences using the PICRUSt2 pipeline.
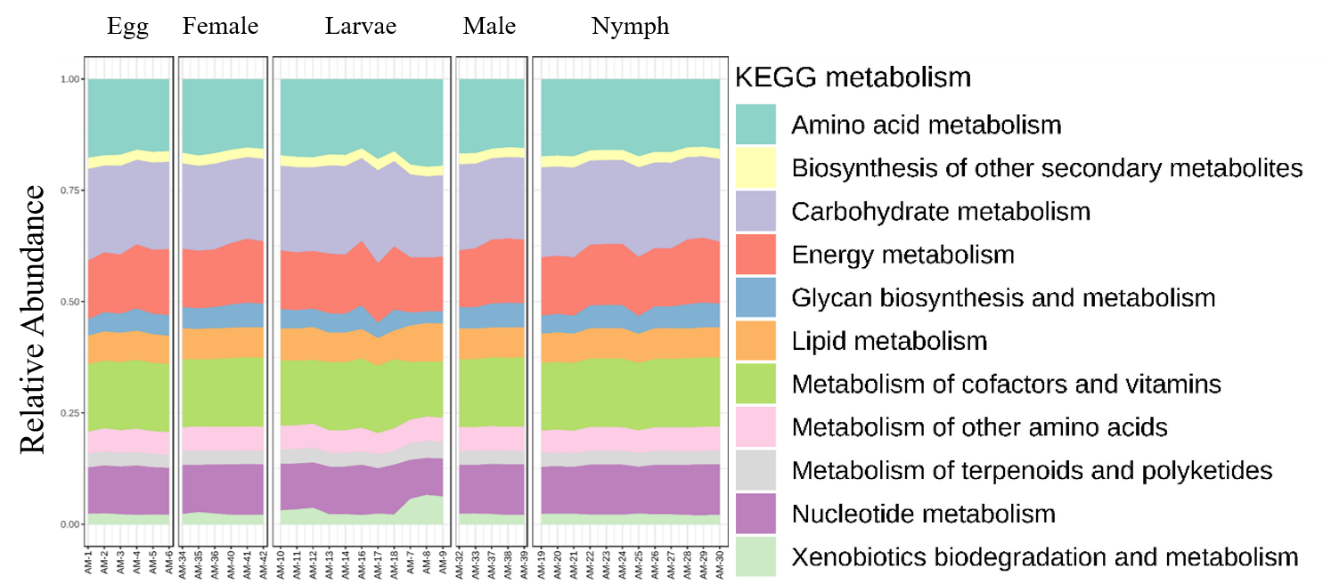


FIGURE S13: **Histogram indicating functional differences in the ovaries of the *Am. maculatum* microbiota** with and without *R. parkeri* infection. KEGG metabolic categories were obtained from 16S rRNA gene sequences using the PICRUSt2 pipeline.
